# Risk of Ischemic Stroke after Intracranial Hemorrhage in Patients with Atrial Fibrillation

**DOI:** 10.1371/journal.pone.0145579

**Published:** 2015-12-23

**Authors:** Michael P. Lerario, Gino Gialdini, Daniel M. Lapidus, Mesha M. Shaw, Babak B. Navi, Alexander E. Merkler, Gregory Y. H. Lip, Jeff S. Healey, Hooman Kamel

**Affiliations:** 1 Department of Neurology, Weill Cornell Medical College, New York, NY, United States of America; 2 Feil Family Brain and Mind Research Institute, Weill Cornell Medical College, New York, NY, United States of America; 3 Centre for Cardiovascular Sciences, University of Birmingham, Birmingham, United Kingdom; 4 Thrombosis Research Unit, Aalborg University, Aalborg, Denmark; 5 Population Health Research Institute, McMaster University, Hamilton, ON, Canada; McGill University Health Center / Royal Victoria, CANADA

## Abstract

**Background:**

We aimed to estimate the risk of ischemic stroke after intracranial hemorrhage in patients with atrial fibrillation.

**Materials and Methods:**

Using discharge data from all nonfederal acute care hospitals and emergency departments in California, Florida, and New York from 2005 to 2012, we identified patients at the time of a first-recorded encounter with a diagnosis of atrial fibrillation. Ischemic stroke and intracranial hemorrhage were identified using validated diagnosis codes. Kaplan-Meier survival statistics and Cox proportional hazard analyses were used to evaluate cumulative rates of ischemic stroke and the relationship between incident intracranial hemorrhage and subsequent stroke.

**Results:**

Among 2,084,735 patients with atrial fibrillation, 50,468 (2.4%) developed intracranial hemorrhage and 89,594 (4.3%) developed ischemic stroke during a mean follow-up period of 3.2 years. The 1-year cumulative rate of stroke was 8.1% (95% CI, 7.5–8.7%) after intracerebral hemorrhage, 3.9% (95% CI, 3.5–4.3%) after subdural hemorrhage, and 2.0% (95% CI, 2.0–2.1%) in those without intracranial hemorrhage. After adjustment for the CHA_2_DS_2_-VASc score, stroke risk was elevated after both intracerebral hemorrhage (hazard ratio [HR], 2.8; 95% CI, 2.6–2.9) and subdural hemorrhage (HR, 1.6; 95% CI, 1.5–1.7). Cumulative 1-year rates of stroke ranged from 0.9% in those with subdural hemorrhage and a CHA_2_DS_2_-VASc score of 0, to 33.3% in those with intracerebral hemorrhage and a CHA_2_DS_2_-VASc score of 9.

**Conclusions:**

In a large, heterogeneous cohort, patients with atrial fibrillation faced a substantially heightened risk of ischemic stroke after intracranial hemorrhage. The risk was most marked in those with intracerebral hemorrhage and high CHA_2_DS_2_-VASc scores.

## Introduction

Atrial fibrillation (AF) increases the risk of ischemic stroke by 3- to 5-fold.[1] Many patients with AF are treated with anticoagulant drugs because of their proven efficacy in preventing thromboembolism.[2, 3] However, anticoagulation with vitamin-K antagonists doubles the risk of intracranial hemorrhage compared to aspirin.[2] Non-vitamin K antagonist oral anticoagulant drugs such as apixaban may not significantly increase the risk of intracranial hemorrhage compared with aspirin,[4] but the use of any antithrombotic therapy in patients with AF is associated with a 0.4% annual risk of intracranial hemorrhage,[4] which is substantial given the high rates of permanent disability and mortality after anticoagulant-associated intracranial hemorrhage.[5]

After the development of intracranial hemorrhage, antithrombotic therapy is almost always stopped for some period of time,[6, 7] if not indefinitely.[8] Given the proven benefit of anticoagulant drugs in reducing stroke, it is likely that the withdrawal of anticoagulation increases the risk of subsequent stroke.[7, 9–11] However, the magnitude of ischemic stroke risk after intracranial hemorrhage in patients with AF remains uncertain because reported rates of AF-related thromboembolism after intracranial hemorrhage vary widely from 2% to 40%.[7, 9, 11] Also, some studies have reported no association between the occurrence of intracranial hemorrhage and an increased risk of subsequent ischemic stroke.[12] No systemic review of the literature has been performed previously in this population. Understanding the risk of ischemic stroke after intracranial hemorrhage is necessary to assess the risks and benefits of resuming anticoagulant therapy[7] or pursuing other therapies such as left atrial appendage closure.[13] Therefore, we evaluated the risk of ischemic stroke after intracranial hemorrhage in a large, heterogeneous cohort of patients with AF.

## Materials and Methods

### Study Design

We performed a retrospective cohort study using administrative discharge data from California, Florida, and New York. Designated agencies in each state collect standardized data on all discharges from nonfederal emergency departments (ED) and acute care hospitals, and report these data in a de-identified format to the Agency for Healthcare Research and Quality for its Healthcare Cost and Utilization Project.[14] A unique linkage number allows patients to be followed across ED visits and hospitalizations over multiple years.[15] Our study was approved by the Weill Cornell Medical College institutional review board.

### Subjects

Using *International Classification of Diseases*, *9* ^*th*^ *Revision*, *Clinical Modification* (*ICD-9-CM*) codes 427.3x in any discharge diagnosis position, we included patients at the time of a first-ever recorded ED visit or hospitalization with an AF diagnosis between 2005–2010 in California, 2005–2011 in Florida, and 2006–2010 in New York. These observation periods were chosen to ensure at least 1 year of follow-up data for all study patients. To focus on incident cases, we excluded patients with a diagnosis of ischemic stroke prior to or during the index visit for AF or a diagnosis of intracranial hemorrhage prior to the index visit for AF. Lastly, to maximize follow-up, we excluded patients who were not residents of California, Florida, or New York.

### Outcomes

Our exposure variable was a time-varying covariate for intracranial hemorrhage, defined as either intracerebral or subdural hemorrhage. We excluded cases of subarachnoid hemorrhage because this type of hemorrhage has a negligible recurrence rate in this population[7] and thus does not present the same long-term management challenge as other types of intracranial hemorrhage. Intracerebral hemorrhage was defined by *ICD-9-CM* code 431 in any hospital discharge diagnosis code position in the absence of a primary discharge code for rehabilitation (V57) or any discharge code for subarachnoid hemorrhage (430) or trauma (800–804 or 850–854). This algorithm has been validated to have a sensitivity of 85% and a specificity of 96% for intracerebral hemorrhage.[16] To our knowledge, *ICD-9-CM* diagnosis codes for subdural hemorrhage have not been previously validated, so we performed an assessment of the reliability of these codes at New York-Presbyterian Hospital/Weill Cornell Medical Center. A board-certified neurologist (A.E.M.) reviewed the medical records of 50 randomly-selected patients with any hospital discharge diagnosis code for subdural hemorrhage (*ICD-9-CM* codes 432.1, 852.2x, or 852.3x) and the records of a random sample of 50 patients with a cerebrovascular diagnosis other than subdural hemorrhage (*ICD-9-CM* codes 430–438 except for 432.1), and adjudicated the presence or absence of subdural hemorrhage while blinded to the diagnosis code. Using this adjudication as the gold standard, we determined that the diagnosis codes for subdural hemorrhage have a sensitivity of 96% (95% confidence interval [CI], 85–99%) and a specificity of 90% (95% CI, 79–97%).

Our outcome was ischemic stroke, defined as *ICD-9-CM* codes 433.x1, 434.x1, or 436 in any hospital discharge diagnosis code position without a concurrent primary discharge code for rehabilitation (V57) or any codes for trauma (800–804 or 850–854), intracerebral hemorrhage (430), or subarachnoid hemorrhage (431). This algorithm has been previously validated to be 86% sensitive and 95% specific for the diagnosis of ischemic stroke.[16]

We similarly used *ICD-9-CM* codes from hospital claims to collect information on the following demographic variables and vascular risk factors that may confound the relationship between intracranial hemorrhage and ischemic stroke: age, sex, race, insurance status, hypertension, diabetes, coronary artery disease, congestive heart failure, peripheral vascular disease, chronic kidney disease, history of transient ischemic attack, and chronic obstructive pulmonary disease. We used this information to calculate each patient’s CHA_2_DS_2_-VASc score, a validated measure of thromboembolic risk in AF.[17]

### Statistical Analysis

Descriptive statistics with exact CIs were used to calculate crude rates of intracranial hemorrhage and ischemic stroke. Kaplan-Meier survival statistics were used to calculate the cumulative incidence of ischemic stroke, stratified by intracranial hemorrhage status (none versus intracerebral versus subdural). Intracranial hemorrhage status was modeled using a time-varying covariate. Patients entered observation at the time of the index encounter with AF and were followed for return visits for ischemic stroke, with the last date of available follow-up being December 31, 2011 in California or New York, and December 31, 2012 in Florida. This resulted in up to 5 years of total follow-up for patients in New York, 6 years in California, and 7 years in Florida. Cox proportional hazards analysis was used to examine the association between intracranial hemorrhage and subsequent ischemic stroke while adjusting for the CHA_2_DS_2_-VASc score. In a sensitivity analysis, we adjusted for all the demographic characteristics and vascular risk factors listed above instead of the CHA_2_DS_2_-VASc score. We visually inspected log-log plots to verify the proportional hazards assumption. Among patients with intracranial hemorrhage, we calculated cumulative rates of stroke stratified by the CHA_2_DS_2_-VASc score. The threshold for statistical significance was set at α = 0.05. Stata/MP version 13 (College Station, TX) was used for all analyses.

## Results

We identified 2,084,735 patients at the time of their first-recorded AF diagnosis. Mean follow-up was 3.2 (±2.1) years for those without intracranial hemorrhage, 2.6 (±2.2) years for those with intracerebral hemorrhage, and 3.2 (±2.1) for those with subdural hematoma. During this time, 50,468 patients (2.4%) developed intracranial hemorrhage. Of these intracranial hemorrhages, 24,330 (48.2%) were intracerebral and 26,138 (51.8%) were subdural. Compared to patients without hemorrhage, those with intracranial hemorrhage were somewhat older and had a slightly higher burden of vascular comorbidities as measured by the CHA_2_DS_2_-VASc score (Table 1).

**Table 1:** Characteristics of Patients with Atrial Fibrillation, Stratified by the Occurrence and Type of Intracranial Hemorrhage.

Characteristic*	Intracerebral Hemorrhage (N = 25,559)	Subdural Hemorrhage (N = 27,799)	No Intracranial Hemorrhage (N = 2,031,377)
Age, mean (SD), y	76.2 (10.9)	78.5 (10.1)	74.6 (13.1)
Female	12,674 (49.6)	11,748 (42.3)	979,342 (48.2)
Race^†^			
White	18,253 (73.0)	21,489 (79.0)	1,592,621 (79.0)
Black	1,812 (7.2)	1,272 (4.7)	122,871 (6.2)
Hispanic	2,724 (10.9)	2,501 (9.2)	170,388 (8.6)
Asian	1,546 (6.2)	1,364 (5.0)	61,386 (3.1)
Other	676 (2.7)	582 (2.1)	44,682 (2.2)
Payment source^‡^			
Medicare	20,569 (80.6)	23,570 (84.7)	1,537,736 (75.7)
Medicaid	1,207 (4.7)	799 (2.9)	80,949 (4.0)
Private	3,023 (11.8)	2,768 (10.0)	333,518 (16.4)
Self-pay	366 (1.4)	281 (1.0)	38,432 (1.9)
Other	391 (1.5)	377 (1.4)	40,487 (2.0)
Hypertension	20,350 (79.6)	20,841 (75.0)	1,466,847 (72.2)
Diabetes	8,167 (32.0)	8,290 (29.8)	606,722 (29.9)
Coronary heart disease	10,471 (41.0)	12,435 (44.7)	890,280 (43.8)
Congestive heart failure	7,815 (30.6)	8,990 (32.3)	717,119 (35.3)
Peripheral vascular disease	2,599 (10.2)	3,132 (11.3)	240,223 (11.8)
COPD	4,473 (17.5)	5,092 (18.3)	485,183 (23.9)
Chronic kidney disease	2,964 (11.6)	3,902 (14.0)	287,552 (14.2)
Transient ischemic attack	752 (2.9)	776 (2.8)	49,616 (2.4)
CHA_2_DS_2_VASc score, median (IQR)^§^	4 (3–4)	4 (3–4)	4 (2–4)
Elixhauser comorbidities, mean (SD) ^||^	2.9 (1.8)	3.0 (1.8)	3.0 (1.9)

Abbreviations: COPD, chronic obstructive pulmonary disease; IQR, interquartile range; SD, standard deviation.

*Data are presented as number (%) unless otherwise specified.

^†^Self-reported by patients or their surrogates. Numbers do not sum to group totals because of missing race/ethnicity data in 1.9% of patients.

^‡^Numbers do not sum to group totals because of missing payment-source data in <0.01% of patients.

^§^The CHA2DS2VASc score assigns 2 points each for age ^3^75 years or prior stroke or transient ischemic attack, and 1 point each for hypertension, diabetes, peripheral vascular disease, age 65–74 years, or female sex. It has been shown to have moderate predictive value for thromboembolism in atrial fibrillation.

^||^Data represent the number of Elixhauser comorbid conditions, which comprise a comprehensive set of 28 comorbidity measures for use with large administrative datasets.

**Fig 1:**
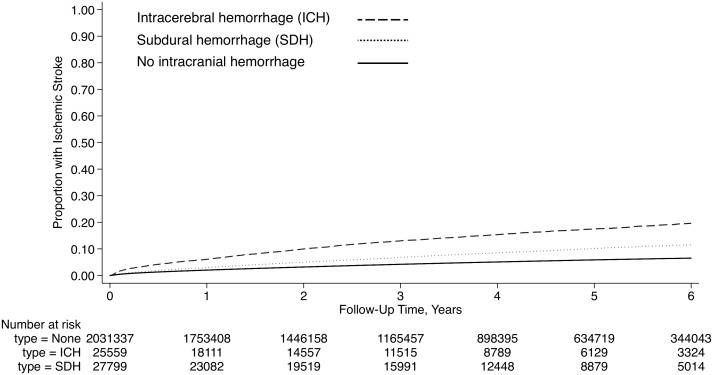
Cumulative ischemic stroke rates in atrial fibrillation patients, stratified by intracranial hemorrhage status. Differences between groups were significant (*P* < 0.001 for test of trend).

During the observation period, 89,594 patients (4.3%) developed ischemic stroke. The 1-year cumulative rate of stroke was 8.1% (95% CI, 7.5–8.7%) after intracerebral hemorrhage, 3.9% (95% CI, 3.5–4.3%) after subdural hemorrhage, and 2.0% (95% CI, 2.0–2.1) in those without intracranial hemorrhage (*P* < 0.001 for test of trend; Fig 1). After adjustment for the CHA_2_DS_2_-VASc score, stroke risk was elevated after both intracerebral hemorrhage (hazard ratio [HR], 2.8; 95% CI, 2.6–2.9) and subdural hemorrhage (HR, 1.6; 95% CI, 1.5–1.7). These associations were essentially unchanged in sensitivity analyses adjusting for the individual components of the CHA_2_DS_2_-VASc score, as well as the additional vascular comorbidities and demographic characteristics, including insurance status, listed in Table 1.

**Fig 2:**
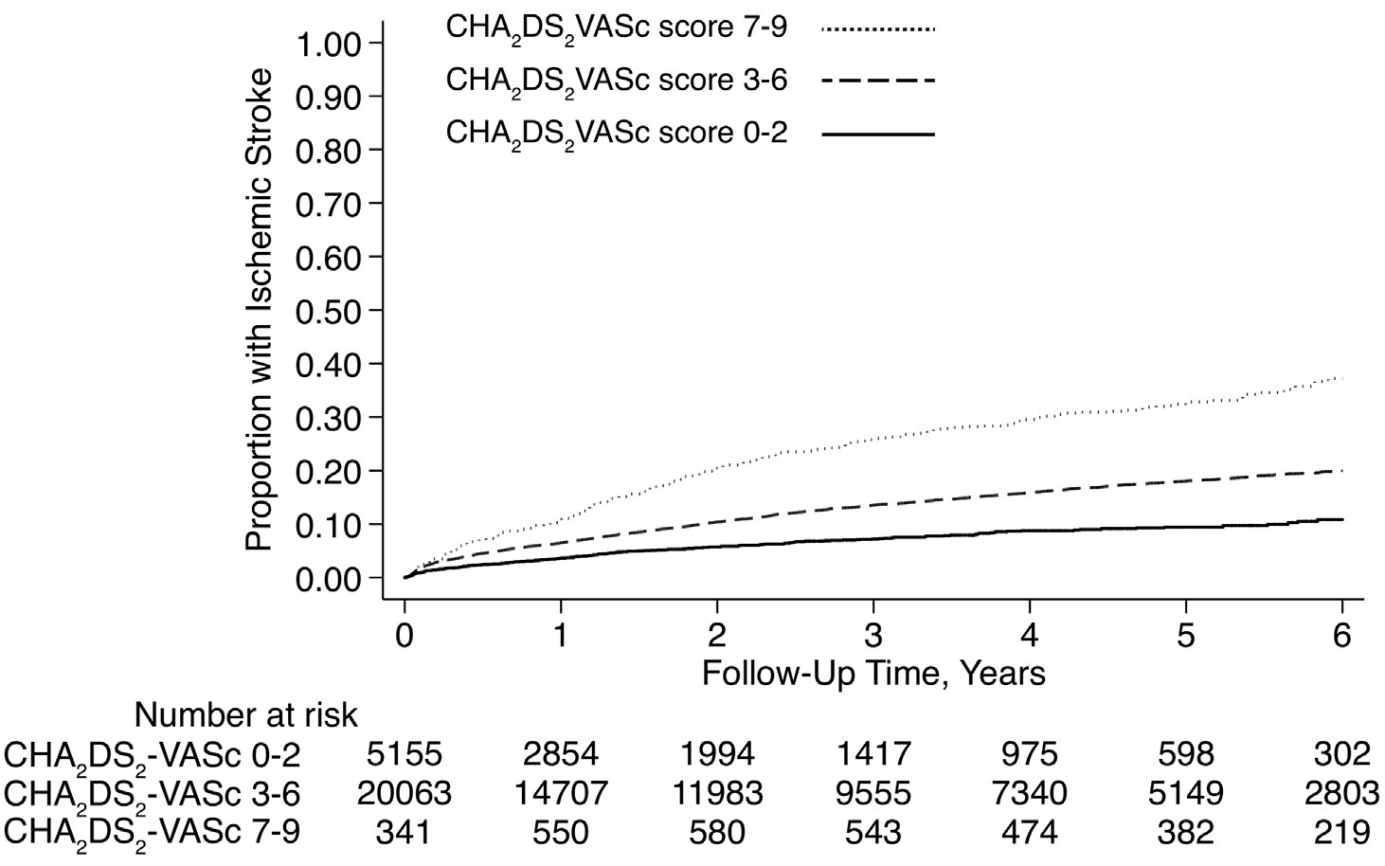
Cumulative ischemic stroke rates after intracerebral hemorrhage in atrial fibrillation patients, stratified by CHA_2_DS_2_-VASc scores. Differences between groups were significant (*P* < 0.001 for test of trend).

**Fig 3:**
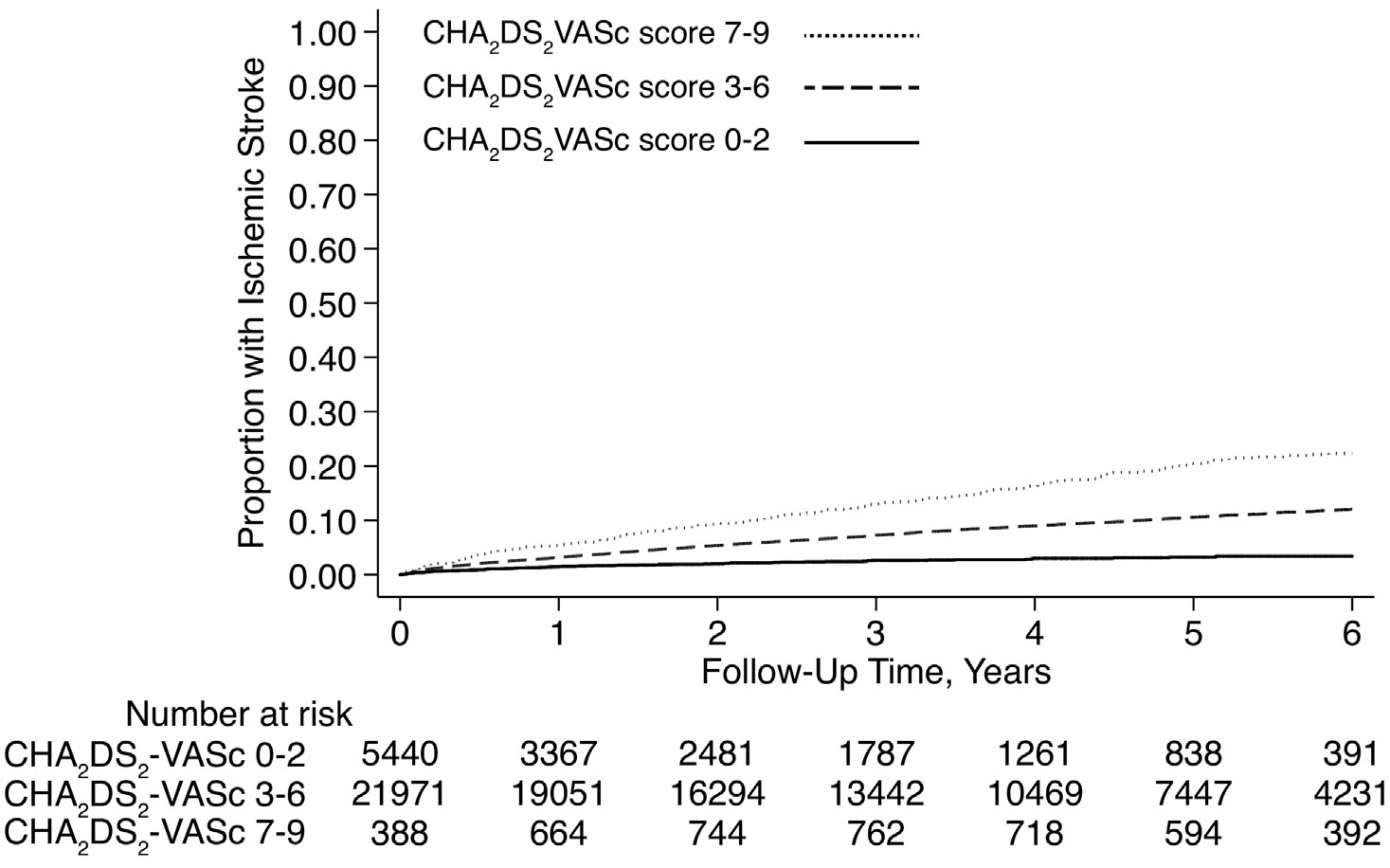
Cumulative ischemic stroke rates after subdural hemorrhage in atrial fibrillation patients, stratified by CHA_2_DS_2_-VASc scores. Differences between groups were significant (*P* < 0.001 for test of trend).

Cumulative rates of ischemic stroke after intracranial hemorrhage varied from 0.9% (95% CI, 0.1–6.0%) in those with subdural hemorrhage and a CHA_2_DS_2_-VASc score of 0, to 33.3% (95% CI, 5.5–94.6%) in those with intracerebral hemorrhage and a CHA_2_DS_2_-VASc score of 9 (Table 2, Figs 2 and 3).

**Table 2:** Cumulative 1-Year Rates of Ischemic Stroke after Intracranial Hemorrhage in Patients with Atrial Fibrillation, Stratified by CHA_2_DS_2_-VASc Scores*.

CHA_2_DS_2_-VASc score	No Hemorrhage (N = 2,031,377)		ICH (N = 25,559)		SDH (N = 27,799)	
	Number	Stroke Rate	Number	Stroke Rate	Number	Stroke Rate
**0**	78,433	**0.2% (0.14–0.21%)**	320	2.6% (0.7–10.2%)	337	0.9% (0.1–6.0%)
**1**	170,926	**0.4% (0.39–0.45%)**	1,376	4.0% (2.5–6.5%)	1,320	1.2% (0.5–3.1%)
**2**	299,341	**0.8% (0.73–0.80%)**	3,459	4.8% (3.6–6.3%)	3,783	2.2% (1.4–3.3%)
**3**	466,911	**1.3% (1.26–1.32%)**	6,657	6.6% (5.6–7.8%)	7,458	3.1% (2.4–3.9%)
**4**	527,986	**2.1% (0.21–0.22%)**	7,808	8.5% (7.4–9.6%)	8,371	3.6% (3.0–4.4%)
**5**	333,784	**3.4% (3.33–3.45%)**	4,246	11.0% (9.5–12.6%)	4,629	5.9% (4.9–7.2%)
**6**	123,854	**4.6% (4.51–4.74%)**	1,352	10.9% (8.6–13.9%)	1,513	5.8% (4.2–7.9%)
**7**	24,891	**6.2% (5.88–6.43%)**	285	9.9% (6.2–15.6%)	310	5.3% (2.7–10.2%)
**8**	4,409	**7.8% (7.14–8.55%)**	48	26.0% (12.8–48.5%)	67	1.6% (0.2–11.1%)
**9**	842	**8.8% (7.26–10.57%)**	8	33.3% (5.5–94.6%)	11	N/A^†^

*The CHA_2_DS_2_-VASc score assigns 2 points each for age ≥75 years or prior stroke or transient ischemic attack, and 1 point each for hypertension, diabetes, peripheral vascular disease, age 65–74 years, or female gender. It has been validated as a clinical prediction rule for thromboembolism in atrial fibrillation.[17]

^†^The rate in this stratum could not be estimated due to insufficient numbers of patients and events.

## Discussion

In a large, heterogeneous sample of patients with AF, we found a heightened risk of ischemic stroke after intracranial hemorrhage. Intracranial hemorrhage was associated with subsequent stroke risk even after adjustment for demographic characteristics and shared vascular risk factors. Cumulative rates of stroke were higher after intracerebral as opposed to subdural hemorrhage and in those with higher CHA_2_DS_2_-VASc scores.

Several prior studies have evaluated ischemic stroke rates after intracranial hemorrhage. These studies reported a wide range of risk for subsequent stroke—ranging from 2–40%—and conflicting findings on whether intracranial hemorrhage was associated with an increased risk of stroke.[7, 9, 11, 12] Our findings in a large, heterogeneous cohort with several years of follow-up indicate that AF patients face a very high absolute risk of ischemic stroke after intracranial hemorrhage, especially intracerebral hemorrhage. These results are confirmed by recent data from Danish registries which demonstrated an increased risk of ischemic stroke following intracranial hemorrhage,[18] particularly if oral anticoagulation was not resumed.[19] However, these analyses were performed in an ethnically homogenous cohort and did not stratify stroke risk based on compartment of hemorrhage (e.g., intraparenchymal versus subdural). Currently, the vast majority of patients do not resume anticoagulation after intracerebral hemorrhage,[19, 20] so our findings may be explained by low rates of proven anticoagulant therapy in this population.

Our study has several noteworthy limitations. Most importantly, we lacked data on antithrombotic drug therapy, and therefore could not assess the risk of ischemic stroke after intracranial hemorrhage in relation to the status of antithrombotic drug use. The use of deidentified data precluded linking of these patient records to Medicare prescription data, which would have allowed adjustment for antithrombotic therapy. However, this would be expected to introduce a conservative bias, because it is likely that at least some proportion of patients resumed anticoagulant therapy after a period of time,[7, 20] and therefore the risk of ischemic stroke after intracranial hemorrhage in patients who are not receiving anticoagulant therapy is likely to be even higher than we found. Future use of registries which do include prescription data could prove helpful in linking stroke rates to the selection and timing of antithrombotic reintroduction following intracranial hemorrhage. Second, we relied on administrative data and may have misclassified patient characteristics and outcomes. We attempted to maximize the reliability of our classifications by using validated codes. Furthermore, misclassifications of intracranial hemorrhage and ischemic stroke would mostly have served to attenuate associations and bias our study towards finding no association between intracranial hemorrhage and ischemic stroke. Nevertheless, we were unable to assess the risk of stroke in relation to certain clinical characteristics, such as the size and precise etiology of intracranial hemorrhage. Lobar intracranial hemorrhages are associated with a higher risk of recurrence than hemorrhages affecting the deeper structures of the brain,[21] and patients with lobar hemorrhages may have been less likely to resume anticoagulant therapy[8] and thus may have faced a higher risk of subsequent stroke. Furthermore, it is possible that some comorbidities were not recorded or coded during the inpatient admissions, and therefore not included in our analysis. However, this would likely underestimate the risk of future ischemic stroke as our patients would have even more vascular comorbidities in actuality than we reported. Third, we lacked outpatient data and therefore likely sampled a sicker cohort than the general population of patients with AF. However, this bias would mostly apply to the control group without intracranial hemorrhage, because intracranial hemorrhage almost always results in hospitalization.[6] Therefore, when compared to the overall population of patients with AF, patients with AF and intracranial hemorrhage may face an even higher relative risk of ischemic stroke than we found.

## Conclusions

AF patients with intracranial hemorrhage faced a heightened risk of subsequent ischemic stroke, and the absolute risks were notably high for those with intracerebral hemorrhage and/or high CHA_2_DS_2_-VASc scores. Our findings may have important clinical implications for this vulnerable group of patients who face a particularly high risk of thromboembolism when anticoagulation is withdrawn after hemorrhage.[7, 9–11] Although recent observational data suggests that timely resumption of anticoagulation may improve outcomes,[19, 22] reintroduction of anticoagulation following intracranial hemorrhage has been uncommon in practice during the same time period.[19, 20] Given the fact that the newer, non-vitamin K antagonist oral anticoagulant drugs cause substantially less intracranial hemorrhage than vitamin K antagonists,[3] further study correlating rates of stroke with antithrombotic usage patterns is needed to better evaluate the risks and benefits of anticoagulant resumption and the implementation of therapeutic alternatives to anticoagulation, such as left atrial appendage closure devices,[13] in patients with high risk of recurrent hemorrhage.

## Supporting Information

S1 Checklist Strobe Statement. Checklist of items that should be included in reports of observational studies.
(DOC)

## References

[1] Wolf PA, Dawber TR, Thomas HE Jr, Kannel WB. Epidemiologic assessment of chronic atrial fibrillation and risk of stroke: the Framingham study. Neurology. 1978;28(10):973–7. Epub 1978/10/01. . 570666 10.1212/wnl.28.10.973

[2] Aguilar MI, Hart R, Pearce LA. Oral anticoagulants versus antiplatelet therapy for preventing stroke in patients with non-valvular atrial fibrillation and no history of stroke or transient ischemic attacks. The Cochrane database of systematic reviews. 2007;(3):CD006186 Epub 2007/07/20. 10.1002/14651858.CD006186.pub2 . 17636831 PMC13591242

[3] Ruff CT, Giugliano RP, Braunwald E, Hoffman EB, Deenadayalu N, Ezekowitz MD, et al Comparison of the efficacy and safety of new oral anticoagulants with warfarin in patients with atrial fibrillation: a meta-analysis of randomised trials. Lancet. 2014;383(9921):955–62. 10.1016/S0140-6736(13)62343-0 . 24315724

[4] Connolly SJ, Eikelboom J, Joyner C, Diener HC, Hart R, Golitsyn S, et al Apixaban in patients with atrial fibrillation. N Engl J Med. 2011;364(9):806–17. Epub 2011/02/12. 10.1056/NEJMoa1007432 . 21309657

[5] Dowlatshahi D, Butcher KS, Asdaghi N, Nahirniak S, Bernbaum ML, Giulivi A, et al Poor prognosis in warfarin-associated intracranial hemorrhage despite anticoagulation reversal. Stroke. 2012;43(7):1812–7. 10.1161/STROKEAHA.112.652065 . 22556194

[6] Morgenstern LB, Hemphill JC 3rd, Anderson C, Becker K, Broderick JP, Connolly ES Jr., et al Guidelines for the management of spontaneous intracerebral hemorrhage: a guideline for healthcare professionals from the American Heart Association/American Stroke Association. Stroke. 2010;41(9):2108–29. Epub 2010/07/24. doi: STR.0b013e3181ec611b [pii] 10.1161/STR.0b013e3181ec611b . 20651276 PMC4462131

[7] Majeed A, Kim YK, Roberts RS, Holmstrom M, Schulman S. Optimal timing of resumption of warfarin after intracranial hemorrhage. Stroke. 2010;41(12):2860–6. Epub 2010/10/30. doi: STROKEAHA.110.593087 [pii] 10.1161/STROKEAHA.110.593087 . 21030703

[8] Eckman MH, Rosand J, Knudsen KA, Singer DE, Greenberg SM. Can patients be anticoagulated after intracerebral hemorrhage? A decision analysis. Stroke. 2003;34(7):1710–6. Epub 2003/06/14. 10.1161/01.str.0000078311.18928.16 . 12805495

[9] Phan TG, Koh M, Wijdicks EF. Safety of discontinuation of anticoagulation in patients with intracranial hemorrhage at high thromboembolic risk. Arch Neurol. 2000;57(12):1710–3. . 11115236 10.1001/archneur.57.12.1710

[10] Broderick JP, Bonomo JB, Kissela BM, Khoury JC, Moomaw CJ, Alwell K, et al Withdrawal of antithrombotic agents and its impact on ischemic stroke occurrence. Stroke. 2011;42(9):2509–14. 10.1161/STROKEAHA.110.611905 21719769 PMC3166233

[11] Jung HS, Jeon IC, Chang CH, Jung YJ. Effect of discontinuation of anticoagulation in patients with intracranial hemorrhage at high thromboembolic risk. Journal of Korean Neurosurgical Society. 2014;55(2):69–72. 10.3340/jkns.2014.55.2.69 24653798 PMC3958575

[12] Goldstein JN, Fazen LE, Wendell L, Chang Y, Rost NS, Snider R, et al Risk of thromboembolism following acute intracerebral hemorrhage. Neurocrit Care. 2009;10(1):28–34. 10.1007/s12028-008-9134-3 . 18810667 PMC4307935

[13] Reddy VY, Sievert H, Halperin J, Doshi SK, Buchbinder M, Neuzil P, et al Percutaneous left atrial appendage closure vs warfarin for atrial fibrillation: a randomized clinical trial. JAMA. 2014;312(19):1988–98. 10.1001/jama.2014.15192 . 25399274

[14] Agency for Healthcare Research and Quality. Healthcare Cost and Utilization Project. http://hcupnet.ahrq.gov. Accessed June 29, 2015.

[15] Agency for Healthcare Research and Quality. HCUP methods series: methodological issues when studying readmissions and revisits using hospital administrative data. http://www.hcup-us.ahrq.gov/reports/methods/2011_01.pdf. Accessed June 29, 2015.

[16] Tirschwell DL, Longstreth WT Jr. Validating administrative data in stroke research. Stroke. 2002;33(10):2465–70. Epub 2002/10/05. . 12364739 10.1161/01.str.0000032240.28636.bd

[17] Lip GY, Nieuwlaat R, Pisters R, Lane DA, Crijns HJ. Refining clinical risk stratification for predicting stroke and thromboembolism in atrial fibrillation using a novel risk factor-based approach: the Euro Heart Survey on Atrial Fibrillation. Chest. 2010;137(2):263–72. Epub 2009/09/19. doi: chest.09-1584 [pii] 10.1378/chest.09-1584 . 19762550

[18] Brønnum Nielsen P, Larsen TB, Gorst-Rasmussen A, Skjøth F, Rasmussen LH, Lip GY. Intracranial hemorrhage and subsequent ischemic stroke in patients with atrial fibrillation: a nationwide cohort study. Chest. 2015;147(6):1651–8. 10.1378/chest.14-2099 . 25412369

[19] Nielsen PB, Larsen TB, Skjøth F, Gorst-Rasmussen A, Rasmussen LH, Lip GY. Restarting Anticoagulant Treatment After Intracranial Hemorrhage in Patients With Atrial Fibrillation and the Impact on Recurrent Stroke, Mortality, and Bleeding: A Nationwide Cohort Study. Circulation. 2015;132(6):517–25. 10.1161/CIRCULATIONAHA.115.015735 . 26059010

[20] Pennlert J, Asplund K, Carlberg B, Wiklund PG, Wisten A, Åsberg S, et al Antithrombotic Treatment Following Intracerebral Hemorrhage in Patients With and Without Atrial Fibrillation. Stroke. 2015 10.1161/STROKEAHA.115.009087 . 26159794

[21] Poon MT, Fonville AF, Al-Shahi Salman R. Long-term prognosis after intracerebral haemorrhage: systematic review and meta-analysis. J Neurol Neurosurg Psychiatry. 2014;85(6):660–7. Epub 2013/11/23. 10.1136/jnnp-2013-306476 . 24262916

[22] Kuramatsu JB, Gerner ST, Schellinger PD, Glahn J, Endres M, Sobesky J, et al Anticoagulant reversal, blood pressure levels, and anticoagulant resumption in patients with anticoagulation-related intracerebral hemorrhage. JAMA. 2015;313(8):824–36. 10.1001/jama.2015.0846 . 25710659

